# Insecticidal Activity of Some Reducing Sugars Against the Sweet Potato Whitefly, *Bemisia tabaci*, Biotype B

**DOI:** 10.1673/031.010.20301

**Published:** 2010-12-07

**Authors:** Jing S. Hu, Dale B. Gelman, Michael E. Salvucci, Yan P. Chen, Michael B. Blackburn

**Affiliations:** ^1^ Invasive Insect Biocontrol and Behavior Laboratory, USDA, ARS, PSI, Beltsville, MD 20705, USA; ^2^ USDA-ARS US Arid-Land Agricultural Research Center, Maricopa, AZ 85138, USA; ^3^ Bee Research Laboratory, USDA, ARS, PSI, Beltsville, MD 20705, USA

**Keywords:** antifeedant, artificial diet, toxic sugars, arabinose, mannose, ribose, xylose

## Abstract

The effects of 16 sugars (arabinose, cellobiose, fructose, galactose, gentiobiose, glucose, inositol, lactose, maltose, mannitol (a sugar alcohol), mannose, melibiose, ribose, sorbitol, trehalose, and xylose) on sweet potato whitefly *Bemisia tabaci* (Gennadius) (Hemiptera: Aleyrodidae) survival were determined using *in vitro* bioassays. Of these sugars, arabinose, mannose, ribose, and xylose were strongly inhibitory to both nymphal and adult survival. When 10% mannose was added to the nymphal diet, 10.5%, 1.0%, and 0% developed to the 2^nd^, 3^rd^, and 4^th^ instars, respectively. When 10% arabinose was added, 10.8% and 0% of the nymphs molted to the 2^nd^ and 3^rd^ instars, respectively. Addition of 10% xylose or ribose completely terminated *B. tabaci* development, preventing the molt to the 2^nd^ instar. With decreasing sugar concentrations the inhibitory effect was significantly reduced. In tests using adults, arabinose, galactose, inositol, lactose, maltose, mannitol, mannose, melibiose, ribose, sorbitol, trehalose, and xylose significantly reduced mean day survival. Mortality rates were highest when arabinose, mannitol, mannose, ribose, or xylose was added to the diet. Mean day survival was less than 2 days when adults were fed on diet containing 10% of any one of these five sugars. When lower concentrations of sugars were used there was a decrease in mortality. Mode of action studies revealed that toxicity was not due to the inhibition of alpha glucosidase (converts sucrose to glucose and fructose) and/or trehalulose synthase (converts sucrose to trehalulose) activity. The result of agarose gel electrophoresis of RT-PCR products of bacterial endosymbionts amplified from RNA isolated from whiteflies fed with 10% arabinose, mannose, or xylose indicated that the concentration of endosymbionts in mycetomes was not affected by the toxic sugars. Experiments in which *B. tabaci* were fed on diets that contained radio-labeled sucrose, methionine or inulin and one or none (control) of the highly toxic sugars showed that radioactivity (expressed in DPM) in the body, in excreted honeydew and/or carbon dioxide, was significantly reduced as compared to controls. Thus, it appears that the ability of insecticidal sugars to act as antifeedants is responsible for their toxicity to *B. tabaci.*

## Introduction

The sweet potato whitefly, *Bemisia tabaci* (Gennadius) (Hemiptera: Aleyrodidae), Strain B [also known as the silverleaf whitefly (Bellows and Perring 1994)] is a polyphagous homopteran that attacks more than 600 different species of plants in both field and greenhouse settings including food, fiber, and ornamental plant species. *B. tabaci* is responsible for billions of dollars of damage in crop losses each year due to its feeding on plant phloem, its ability to transmit plant pathogenic viruses, and its production of honeydew that is sticky and supports the growth of sooty mold (Gill, 1992; Zalom et al. 1995; Heinz 1996; Henneberry et al. 1997; Henneberry et al. 1998; Chu and Henneberry 1998). Heavy application of pesticides to control *B. tabaci* has led to the development of pesticide resistance and has caused the decline of *B. tabaci* natural enemies (Horowitz and Ishaaya 1995; Cahill et al. 1996a, 1996b; Cisneros and Mujica 1998). Therefore, finding environmentally friendly biological agents for farmers and greenhouse growers to use to cost-effectively control *B. tabaci* is a top priority.

The nutritional requirements of *B. tabaci* are provided by the plant phloem on which they feed. Phloem has a very high carbohydrate content (Fisher and Gifford 1986; Winter et al. 1992), typically in the form of the disaccharide sucrose (Zeigler 1975) which satisfies their energy needs. *B. tabaci* convert sucrose to glucose and fructose, monosaccharides that support growth and development or are excreted in the honeydew [as summarized in Salvucci (2000)]. The identity and concentration of the carbohydrates in honeydew are influenced by the sucrose concentration in the diet (Salvucci et al. 1997). Excess sucrose is converted to the disaccharide trehalulose which can be present in even higher concentrations than glucose and fructose in honeydew produced by *B. tabaci* (Byrne and Miller 1990; Hendrix et al. 1992). Salvucci et al. (1997) showed that the amount of trehalulose synthesized and excreted depends on the concentration of dietary sucrose and the relative activities of trehalulose synthase (promotes the isomerization of sucrose to trehalulose) and sucrase (promotes the hydrolysis of sucrose to glucose and fructose). Under conditions of heat stress, *B. tabaci* produces unusually large amounts of sorbitol from sucrose (Salvucci et al. 1999)

Results from experiments designed to develop improved artificial diets for *B. tabaci* revealed that some simple sugars had insecticidal effects (unpublished results). Importantly, it has been reported that the genes that regulate the synthesis of some of these insecticidal sugars are present in plants (Burget et al. 2003; Kawasaki 1981; Conklin et al. 1999; Hornung-Leoni 2007). Therefore, experiments were conducted to evaluate the effects of 16 selected sugars for toxicity against *B. tabaci.* Here the results of these experiments and of additional studies to determine the mode(s) of action of the insecticidal sugars are described. In mode of action studies, it was important to compare the effects of non-insecticidal sugars with those that were toxic to *B. tabaci* and to compare relative toxicity (highly, moderately, slightly, or non toxic) to uptake of radio-label and production of carbon dioxide.

## Materials and Methods

### Chemicals

Sugars were purchased from Sigma Aldrich (www.sigmaaldrich.com) and FreAmine III from B. Braun Medical (www.bbraun.com). Radio-labeled U-^14^C sucrose, L-[methyl-^14^H] methionine, and inulin [^14^C]-carboxylic acid were obtained from Amersham (www.apbiotech.com). The scintillation cocktail, Ecoscint A, was purchased from National Diagnostics (nationaldiagnostics.com).

### Insect Rearing

Whiteflies were reared on a variety of plants, including green bean cv. Roma II (Burpee, www.burpee.com), sweet potato, tomato cv. Bush Big Boy (Burpee), cotton cv. Stoneville ST 474 (Stoneville Pedigreed Seed Co., Maricopa, AZ, USA), collard cv. Champion (Meyer Seed Co., www.meyerseedco.com) poinsettia cv. Freedom Red (Paul Ecke Ranch, Encinitas, CA, USA), and eggplant cv. Millionaire Hybrid (Burpee) as described in Gelman et al. (2002, 2005). The *B. tabaci* colony was maintained in a walk-in, climate-controlled insect growth chamber (26 ±± 2°°C, L:D 16:8, and RH of 60–80%).

### *In vitro* rearing of *B. tabaci* nymphs and adults

**Figure 1.**
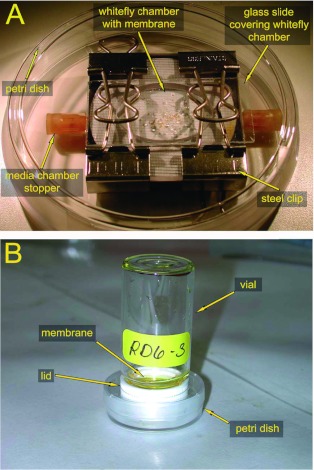
Nymphal (A) and adult (B) rearing chambers. The nymphal rearing chamber was designed by Jancovich et al. (1997). The adult rearing chamber was modeled after the one described in Salvucci and Crafts-Brandner (2000). High quality figures are available online.

Nymphal rearing chambers (Figure 1A) were assembled and eggs (6 days after deposition) were collected from leaves, cleaned, and placed on the membranes of the rearing chambers according to the methods described by Jancovich et al. (1997). Rearing chambers were maintained in a desiccator at a temperature of 26±±2°°C, RH of 75%, and a photoperiodic regimen of L:D 16:8. Nymphal development was monitored with a stereoscopic microscope and nymphs were identified according to instar (Gelman et al. 2002). *B. tabaci* were monitored on day 25, a time when adults would have completed emergence. Adult emergence was tallied by counting the exuviae from which the adults had emerged, and percent survival was calculated by dividing the number of exuviae by the number of 1^st^ instars that had been observed 3 days after the eggs were placed on the membrane. When percent survival for each instar was calculated, the number of living 2^nd^, 3^rd^, and 4^th^ instars was determined and that number was divided by the number of individuals that had hatched to the 1^st^ instar.

For rearing adults, the *in vitro* rearing system described by Salvucci and Crafts-Brandner (2000) was modified (Blackburn et al. 2005) to monitor mean day survival of *B. tabaci* fed on artificial diets containing various sugars. Adults were anaesthetized with CO_2_ and placed in rearing chambers (Figure 1B) that were maintained in an incubator under conditions of 24±±2°°C, RH of 40–60%, and L:D 16:8. To facilitate counting, the initial number of *B. tabaci* was determined approximately 18 h after they were placed in the chamber and the number living adults was counted daily for the next 10 days. Mean day survival was calculated at the end of the 10-day period.

Nymphal and adult control diets were made up in distilled water and contained 15% sucrose and 5% Difco yeast extract (nymphs) or 15% sucrose and 10% FreAmine III (adults). For test diets, sugars (10% final concentration) were added individually so as to maintain the concentrations of sucrose and yeast or FreAmine III present in the control diet. All diets were sterilized by passing them through a 0.22 µµm filter (Millipore, www.waters.com). Adult and nymphal feeding chambers contained 1.5 ml of diet.

### Effect of toxic sugars on enzyme activity in whole body extracts of adult *B. tabaci*

Adults were fed on artificial diet (controls) or on diet containing one of the toxic sugars (5%). After 24 h, living adults were collected, frozen at -20°°C for 2 h, and shipped on dry ice to the Western Cotton Research Laboratory in Phoenix, AZ. Alpha glucosidase and trehalulose synthase activity in supernatants of homogenized and centrifuged *B. tabaci* preparations were determined as described in Salvucci et al. (1997). Briefly, adults were extracted in 50 mM potassium phosphate, pH 6.5, 5.0 mM dithiotreitol, and 1% Triton X-100. Alpha-glucosidase and trehalulose synthase activities were assayed at 30°°C in reaction mixtures containing 0.1 M potassium phosphate, pH 6.5, 420 mM sucrose, and 330 mM of the indicated sugar. After 30 min of incubation the reaction mixture was boiled for 2 min to stop the reaction and the tubes were immediately centrifuged. The amount of glucose, fructose, and trehalulose in the supernatants was determined using anion-exchange HPLC.

### Effect of toxic sugars on the survival of *B. tabaci* endosymbionts

In order to determine if sugars could cause a reduction or elimination of endosymbionts in *B. tabaci*, adults were fed on artificial diet (controls) or on artificial diet containing 10% arabinose, mannose, or xylose. After 48 h, living *B. tabaci* from each of the four groups were collected by aspiration and transferred to eppendorf tubes. They were frozen immediately at -20°°C for subsequent molecular analysis. While frozen they were ground to a powder and homogenized in 500 µµl of TRIzol Reagent (RNA extraction kit, Invitrogen, www.invitrogen.com) for RNA extraction according to the manufacturer's instructions. After isopropanol precipitation, RNA pellets were resuspended in DEPC-treated water in the presence of Ribonulcease Inhibitor (Invitrogen) and stored at -80°°C for further analysis. RNA concentration was measured at an absorbance of 260 nm.

The Access RT-PCR system (Promega, www.promega.com) was used to perform the RT-PCR reaction to examine the presence and survival of endosymbionts in the *B. tabaci* samples after treatment with the toxic sugars. PCR primers specific for 16S rRNA —— encoding DNA sequences of the primary endosymbionts of *B. tabaci*, (28F: 5′?-TGCAAGTCGAGCGGCATCAT-3′? and 1098R: 5′?-AAAGTTCCCGCCTTATGCGT-3′?) [based on Zchori-Fein and Brown (2002)] were used to amplify a 1000-bp PCR fragment. Four 10-fold serial dilutions (10ng, 1 ng, 0.1 ng, and 0.01 ng) of RNA for each sample were used for the PCR amplification. The RT-PCR Amplification was performed in a total volume of 25 µµl of reaction mixture containing 1X AMV/Tfl reaction buffer, 0.2mM each dNTP, 1.0µµM of each primer, 2.0mM MgSO_4_, 0.1unit AMV reverse transcriptase, and 0.1 unit Tfl DNA polymerase. The RT-PCR was performed under the following conditions: one cycle at 48°°C for 45 min; one cycle of 95°°C for 2 min; 30 cycles at 95°°C for 30 sec, 55°°C for 1 min, and 72°°C for 1 min; one cycle of 72°°C for 10 min. Amplification products were analyzed by electrophoresis using 1% agarose gels containing 0.5 µµg/ml ethidium bromide and viewed under UV light. PCR amplification was repeated in triplicate, with each replicate performed on a different day.

### Effect of toxic sugars on the uptake of radio-labeled sucrose, methionine, and inulin and the production of CO_2_ by adult *B. tabaci*

**Figure 2.**
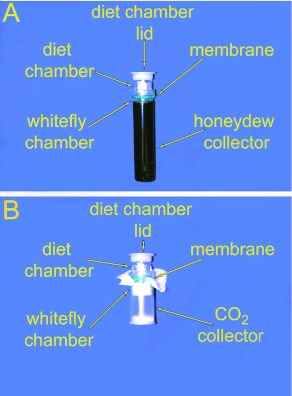
Micro-feeding chambers for measuring *Bemisia tabaci* uptake of radiolabeled sucrose, methionine, and inulin (A) and for measuring B. *tabaci* production of CO_2_ (B). High quality figures are available online.

Micro-concentrators (Amicon, W. R. Grace & Co., www.millipore.com) were adapted to make capped adult micro-feeding chambers having a 0.8 cm^2^ feeding surface composed of a very thin layer of parafilm (Figure 2A). The filtrate collecting tube was used to hold *B. tabaci* and to collect the honeydew. The total volume of diet in each experimental or control feeding chamber was 200 µµl. The diet contained 15% sucrose and 10% FreAmine III (controls) plus 10% of the test sugar (experimental samples). One——four µµci of a radioactive tracer in the form of [U- ^14^C] sucrose, L-[methyl-^3^H] methionine, or inulin-[^14^C]-carboxylic acid were present in the 200 µµl of test diet. Young adults were collected using an aspirator, anaesthetized with CO_2_ and then transferred to the collecting tubes of the feeder (approximately 100–150 *B. tabaci/*tube). They were maintained in an incubator set to provide a temperature of 24±±1°°C, RH of 40–60%, and a photoperiodic regimen of L:D 16:8. Approximately two hours later, after they had begun to feed through the membrane, the collecting tube was replaced by a new one. This was done in order to eliminate excess *B. tabaci* and contribution to the results of the honeydew produced by *B. tabaci* that had not imbibed the test sugars. *B. tabaci* were allowed to feed for 24 h and then the feeder was chilled at -20°°C for approximately 3 min in order to immobilize them. They were removed from the parafilm and the collecting tube, and after the total number of *B. tabaci* was determined, they were placed into a plastic scintillation vial (Snap Cap Bio-Vial, Beckman Instruments, www.beckmancoulter.com) that contained 3 ml of Ecoscint A. They were ground with a glass pestle. Honeydew was recovered by rinsing the collecting tube twice with 250 µµl of distilled water and then transferring the water to a vial that contained 2.5 ml of Ecoscint A. Radioactivity, expressed in DPM, was determined using a Beckman 5801 Liquid Scintillation Counter.Two µµci of [U- ^14^C] sucrose was also used as the radioactive tracer to measure *B. tabaci* respiration. *B. tabaci* were placed in feeders (Figure 2B) as described above and 250 µµl of 1M KOH was placed in the CO_2_ trap (contained a mini stirrer bar) in order to absorb the CO_2_ produced by the *B. tabaci.* After 24 h of feeding, they were immobilized by refrigeration and removed from the feeding chamber. The KOH solution was transferred to a vial containing 2.75 ml of Ecoscint A and radioactivity (in DPM) was determined.

### Statistical Analysis

Data was analyzed using ANOVA. When F-tests were significant, the Tukey HSD Comparison of Means Test was used to analyze for significant differences among the various groups (α? = 0.05). When more than 10 groups were compared, the Bonferroni correction (α? = 0.05/group number) was used in order to control the probability of a Type 1 α? error (falsely rejecting the null hypothesis) since one out of every 20 hypothesis-tests is expected to be significant at the α? = 0.05 level just due to chance (Wikipedia, the free encyclopedia, 2010). However, the Bonferroni correction also increases the probability of a Type 2 error (falsely accepting the null hypothesis) (Wikipedia, the free encyclopedia, 2010), and therefore, it was not used when 10 or fewer groups were being compared.

**Figure 3.**
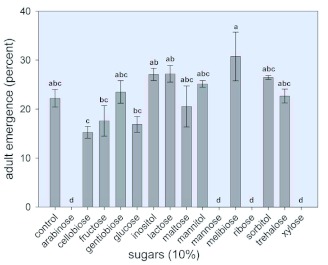
Effect of selected sugars on the survival of *Bemisia tabaci* nymphs. Eggs were placed on the membrane of each nymphal rearing chamber (Figure 1A) and maintained in a desiccator at 26——2°°C, RH of 75% and 16:8 L:D. Upon hatching, nymphs fed on the artificial diet provided. Control diet contained sucrose (15%) and Difco yeast extract (5%). For test diets, 10% of the test sugar was added to the control diet. On day 25 after hatching adult emergence was tallied by counting the exuviae from which the whiteflies had emerged. Each bar represents the mean —— S. E. of at least 5 separate determinations. Means having the same letter designation were not significantly different. High quality figures are available online.

## Results

### Effect of selected sugars on the survival of *B. tabaci* nymphs

**Figure 4.**
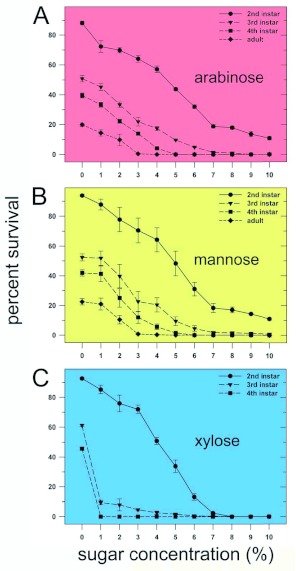
Effect of the concentration of selected sugars on the development of *Bemisia tabaci* nymphs. *B.* *tabaci* were reared under the conditions described in Figure 3. Dose- response curves were generated to measure the effects of arabinose (A), mannose (B), and xylose (C) on the percent of 1^st^ instars that molted to 2^nd^, 3^rd^, and 4^th^ instars and to adults. Each value represents the mean ±± S. E. of at least 5 separate determinations. High quality figures are available online.

Among the 15 sugars tested, only arabinose, mannose, ribose, and xylose were highly insecticidal against nymphs (Figure 3). At a concentration of 10%, treatment with these four sugars resulted in zero percent adult emergence. Therefore, dose-response curves that measured percent survival of 2^nd^, 3^rd^, and 4^th^ instars and of adults for arabinose, mannose, and xylose at concentrations of between 0 and 10% were generated (Figure 4). For arabinose, mannose, and xylose, the values for the percent sugar concentration that resulted in a 50% reduction (as compared to control values) in the 1^st^ to 2^nd^ instar molt were approximately 5% (Figure 4A), 5% (Figure 4B), and 4% (Figure 4C), respectively. At a concentration of 8%, xylose completely inhibited the development to the 2^nd^ instar (Figure 4C), while even at a concentration of 10%, neither arabinose nor mannose was able to prevent the appearance of 2^nd^ instars. At a concentration of 7.0% xylose, no 3^rd^ instars were observed, and at a concentration of 9 and 10%, respectively, arabinose (Figure 4A) and mannose (Figure 4B) completely inhibited the 2^nd^ to 3^rd^ instar molt, although percent survival was very close to zero at a concentration of 7% for these two sugars. Importantly, while the three sugars effectively prevented the 2^nd^ to 3^rd^ instar molt at a concentration of 8%, xylose, when present at a concentration of only 1%, reduced the percent of the 2^nd^ to 3^rd^ instar molt to 9%. Arabinose and mannose required a concentration of 5% to effect the same reduction to 9% in this molt. The percent concentration of arabinose, mannose, and xylose that completely prevented the 3^rd^ to 4^th^ instar molt was 5.0%, 6.0%, and 1.0%, respectively. These results clearly demonstrate that xylose is more toxic to *B. tabaci* nymphs than either arabinose or mannose. A dose-response curve that measured the percent survival of 2^nd^ instars was also generated for ribose (results not shown). At doses of 5% and 7.5%, respectively, only 2.4% and 0% reached the 2^nd^ instar, whereas at a dose of 5% xylose or mannose, approximately 30% achieved the molt to the 2^nd^ instar, and at a dose of 5% arabinose, more than 40% molted to the 2^nd^ instar. Therefore, of the four sugars, ribose appears to be the most toxic to *B. tabaci* nymphs.

### Effect of selected sugars on the survival of *B. tabaci* adults

**Figure 5.**
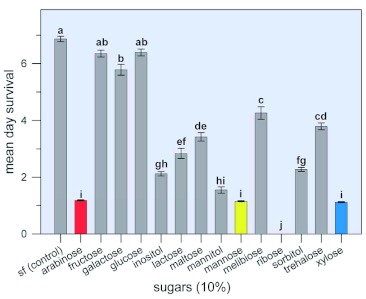
Effect of selected sugars on the survival of *Bemisia tabaci* adults. Adults were anaesthetized with CO_2_ and placed in rearing chambers (Figure 1B) in an incubator at 24±±2°°C, RH of 40 – 60% and 16:8 L:D. Control diet contained sucrose (15%) and FreAmine III (10%). For test diets, 10% of the test sugar was added to the control diet. Each bar represents the mean ±± S. E. of at least 10 separate determinations. Means having the same letter designation were not significantly different. High quality figures are available online.

**Figure 6.**
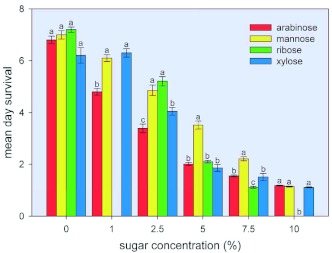
Effect of sugar (arabinose, mannose, ribose, and xylose) concentration on mean day survival of *Bemisia tabaci* adults. Adults were reared under the conditions described in Figure 5. Dose-response curves were generated to measure the effects of arabinose, mannose, ribose, and xylose on mean day survival. The effect of 1% ribose was not determined. Each value represents the mean ±± S. E. of at least 6 separate determinations. For each concentration, means having the same letter designation were not significantly different. High quality figures are available online.

Among the 14 sugars tested (concentration = 10%), arabinose, mannose, mannitol, ribose, and xylose elicited a mean day survival of 1.19, 1.15, 1.55, 0, and 1.12 days, respectively, for adults as compared to the control value of 6.8 days (Figure 5). Other sugars that exhibited significant insecticidal effects were galactose (significant but minimal), inositol, lactose, maltose, melibiose, sorbitol, and trehalose. Dose-response curves were generated for four of the most potent sugars (arabinose, mannose, ribose, and xylose) at concentrations of between 0 and 10%, at intervals of 1.0, 2.5, 5.0, 7.5, and 10% except for ribose for which intervals were set at 2.5, 5.0, 7.5, and 10% (Figure 6). For each sugar, a one way ANOVA followed by a one-sided Dunnett's Multiple Comparisons test with the control was performed on mean day survival values in the control group and in the groups exposed to the various concentrations of the sugar to determine at what concentration of the test sugar a significant drop in mean day survival was first observed (results not shown). Mean day survival was first observed to decrease at a sugar concentration of 1.0% for arabinose and mannose, and at a sugar concentration of 2.5% for xylose. When the order of toxicity of the various test sugars was evaluated, it was found that the order of toxicity varied according to the percent concentration of the test sugar. Thus, at a 2.5% sugar concentration, toxicity from greatest to least was arabinose > xylose > mannose = ribose. At a 5% sugar concentration, the order was arabinose = ribose = xylose > mannose; at a 7.5% concentration, ribose > arabinose = xylose > mannose; and at a 10% concentration, ribose > arabinose = mannose = xylose. The concentration of arabinose, xylose, ribose, or mannose required to reduce the mean day survival to 50 percent of control insects was 2.5%, 3.5%, 3.7%, and 5.0%, respectively. Based on this parameter, the toxicity of the four sugars from greatest to least is: arabinose > xylose = ribose > mannose.

### Effect of arabinose, mannose and xylose on α?-glucosidase and trehalulose synthase activity in adult whiteflies

**Figure 7.**
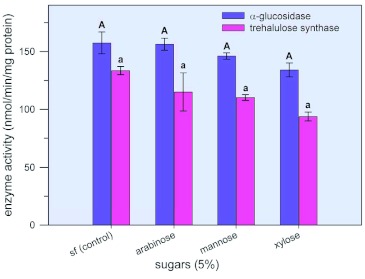
Effects of arabinose, mannose, and xylose on α?-glucosidase (catalyzes the conversion of sucrose to glucose and fructose) and trehalulose synthase (catalyzes the isomerization of sucrose to trehalulose) activity in adult *Bemisia tabaci.* Adults were reared under the conditions described in Figure 5. However, for test diets, 5% of the test sugar was added to the control diet. After 24 h, living whiteflies were collected, frozen at -20°°C, and shipped on dry ice to the Western Cotton Research Lab (Phoenix AZ) where enzyme activity was determined from cell-free *B.* *tabaci* extracts. The assay was performed at 30°°C in reaction mixtures containing 0.1 M postassium phosphate, pH 6.5, 420 mM sucrose, and 330mM of the indicated sugars in a total volume of 50 ul. The amount of glucose, fructose, and trehalulose produced in the reactions was determined using anion-exchange HPLC. Each value represents the mean ±± S. E. of at least 4 separate determinations. For each enzyme, means with the same letter designation were not significantly different. High quality figures are available online.

After treatment with arabinose, mannose, or xylose for 24h, activity for both α?-glucosidase and trehalulose synthase assayed in the presence of each sugar was not significantly different for experimentals and controls (Figure 7).

### Effect of arabinose, mannose, and xylose on the endosymbiont populations present in adult *B. tabaci*

**Figure 8.**
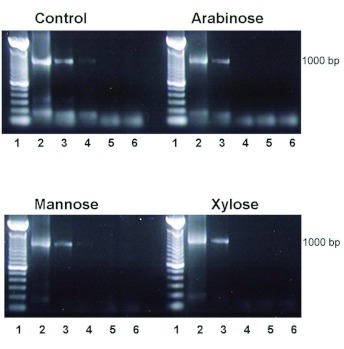
Effects of arabinose, mannose, and xylose on endosymbiont survival of adult *Bemisia tabaci.* Adults were reared under the conditions described in Figure 5. PCR was used to determine the presence of the 16S ribosomal RNA or (cDNA of 16S rDNA) of bacterial endosymbionts that were present in *B.* *tabaci* treated with arabinose, mannose, or xylose and in control samples that did not contain a toxic sugar. The higher the level of input of total RNA, the greater the intensity of the PCR band. Values on the X axis (1–6) represent standards and 10-fold dilutions (beginning with 10 ng), respectively. For each toxic sugar and the control, a representative run is shown. The detection limit for controls and for experimentals fed with the toxic sugars was determined. High quality figures are available online.

PCR bands corresponding to the presence of the 16S ribosomal RNA or (cDNA of 16S rDNA) of bacterial endosymbionts were present in controls and in those supplied with each of the toxic sugars (Figure 8). The higher the level of input of total RNA, the greater the intensity of the PCR band. The limit for significant detection of bacterial endosymbionts was 1 ng of total RNA extracted from *B. tabaci* fed with mannose, arabinose, and xylose. Controls had a similar detection limit for endosymbionts. Thus, there was no significant difference in the concentration of the 16S ribosomal RNA in the control and the three experimental groups.

### Effect of toxic sugars on the uptake of radio-labeled sucrose, methionine and inulin and the production of CO_2_ by adult *B. tabaci*

**Figure 9.**
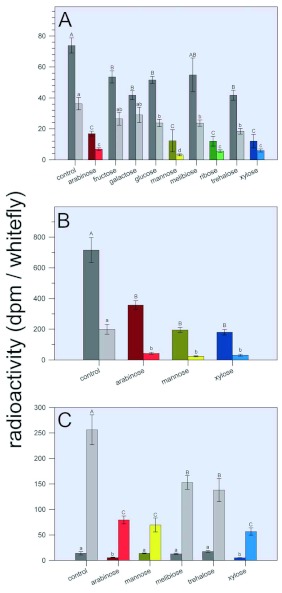
Effect of selected sugars on the uptake of radio-labeled sucrose (A), methinone (B), and inulin (C). Anaesthetized adults were placed in micro-feeding chambers (Figure 2A) as described in Materials and Methods. [U- ^14^C] sucrose (2 µµCi/200 µµl of diet), L-[methyl- ^3^H] methionine (4 µµCi/200 µµl of diet) or inulin-[^14^C]-carboxylic acid (1 µµCi/200 µµl of diet) was added to the artificial diet in the feeding chamber. After 24 h, the amount of radioactivity in homogenized *Bemisia* *tabaci* and in honeydew was determined. Radioactivity is expressed as dpm/*B.* *tabaci.* Each bar represents the mean ±± S. E. of at least 5 separate determinations. For each pair of bars, the 1st bar represents DPM for the adult body and the 2^nd^ bar represents DPM for the *B.* *tabaci* honeydew. For each graph, means having the same upper or lower case letter designations were not significantly different. High quality figures are available online.

In order to determine if sugars toxic to adults inhibited feeding, non- insecticidal fructose and glucose, minimally insecticidal galactose, moderately insecticticdal melibiose and trehalose, and highly insecticidal arabinose, mannose, ribose, and xylose (each at a concentration of 10%) were tested for their effects on the uptake of radio-labeled sucrose as compared to controls (Figure 9A). There was a 26–43% reduction in the amount of radioactive label found in bodies when they were fed on diets containing radio-labeled sucrose, and 10% fructose, galactose, glucose, melibiose or trehalose for 24 h as compared to controls, although melibiose did not result in a statistically significant decrease in uptake as compared to controls. When fed on diets containing the highly insecticidal sugars arabinose, mannose, ribose or xylose for 24 h, the amount of radioactive label found in bodies was reduced by 77–84% as compared to controls. When fed on diets containing radio-labeled sucrose and fructose or galactose, there was a small (19–28%), but not statistically significant, reduction in the amount of radioactive label found in honeydew as compared to controls (Figure 9A). The addition of glucose, melibiose or trehalose to the diet resulted in a 33–50% reduction in the amount of radio-label detected in honeydew while the addition of arabinose, mannose, ribose or xylose to the diet resulted in an 81–92% reduction in the amount of radio-label detected in honeydew as compared to controls.

When adults were fed for 24 h on diets containing radio-labeled methionine and 10% arabinose, mannose, or xylose, the amount of radio-label found in both bodies and in honeydew was also significantly reduced (Figure 9B). Arabinose, mannose, and xylose effected a 50, 73, and 75% reduction, respectively, in the DPM detected in bodies and a 78, 84, and 88% reduction, respectively, in the DPM detected in honeydew as compared to controls. When *B. tabaci* were fed on diets containing radio-labeled inulin and 10% arabinose, mannose or xylose there was a significant reduction (69, 73, and 78%, respectively) in the DPM detected in honeydew as compared to controls. When melibiose or trehalose was added to the diet, there was also a significant reduction in the DPM detected in honeydew as compared to controls, but DPM were reduced by only 40 and 46%, respectively. As compared with honeydew, the radio-label detected in bodies was very low as would be expected since inulin is not metabolized and is too large a molecule to pass into the hemolymph from the gut. However, despite the small amount of radio-label detected in the body, arabinose and xylose significantly reduced this amount by approximately 68% as compared to controls, while mannose, melibiose, and trehalose did not elicit a statistically significant reduction.

**Figure 10.**
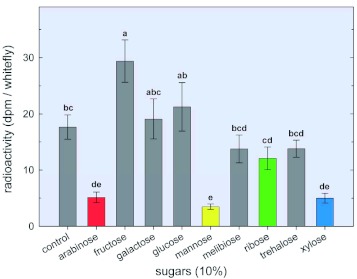
Effect of selected sugars on the production of CO_2_ by adult *Bemisia* *tabaci.* Anaesthetized adults were placed in micro-feeding chambers (Figure 2B) as described in Materials and Methods. Diets contained 10% of the test sugars. [U- ^14^C] sucrose (2 µµCi/200 µµl of diet) was added to the artificial diet in the feeding chamber. After 24 h, the amount of radioactivity in the KOH contained in the CO_2_ trap was determined. Radioactivity is expressed as DPM/individual. Each bar represents the mean ±± S. E. of at least 6 separate determinations. Means having the same letter designations were not significantly different. High quality figures are available online.

To determine the effects of arabinose, fructose, galactose, glucose, mannose, melibiose, ribose, trehalose, and xylose on respiration in adult whiteflies, diets containing radio-labeled sucrose, and 10% of each of these sugars individually, were fed to whiteflies for 24 h and radioactivity of the released and trapped CO_2_ was measured. Only arabinose, mannose and xylose significantly reduced the production of radio-labeled CO_2_ (by 71, 80 and 72%, respectively) as compared to controls (Figure 10). The addition of fructose significantly increased (by 66%) the released and trapped labeled CO_2_ as compared to controls.

## Discussion

The results of this study demonstrate that four sugars: arabinose, mannose, ribose, and xylose, when added to the diet at concentrations of only 1–3% (depending upon the sugar and *B. tabaci* stage), are insecticidal to nymphs and adults. Certain sugars had insecticidal effects that were limited to either immatures or adults. The sugar alcohol mannitol was toxic only to nymphs, while inositol, lactose, maltose melibiose, sorbitol, and trehalose significantly decreased the mean day survival of adults but had no effect on nymphs. Based on dose-response curves, the order of the four highly insecticidal sugars from most to least toxic to nymphs is ribose > xylose > arabinose = mannose. For adults, the order varies according to the percent concentration of the test sugar. However, based on the concentration of arabinose, xylose, ribose or mannose required to reduce the mean day survival as compared to controls to 50 percent, the toxicity of the four sugars from greatest to least is: arabinose > xylose = ribose > mannose. Additional research is needed to explain why mannitol exhibits such a different effect in nymphal and adult stages, why inositol, lactose, maltose, melibiose, sorbitol, and trehalose exhibited some toxicity in adults but not in nymphs, and why the relative toxicities of the four sugars are different in nymphs and adults.

Sugar esters, which are produced naturally by trichomes of Solanaceous plants, and synthetically by reacting sugars with fatty acids, have been reported to be insecticidal for whiteflies, aphids, thrips, psyllids, lepidopteran larvae, and mites (Parr and Thurston, 1968; Hawthorne et al. 1992; Neal et al. 1994, Puterka and Severson 1995; Liu et al. 1996; Chortyk et al. 1996; Sheppard et al. 2003; Stanghellini et al. 2005). The sugar esters are contact insecticides with rapid knock-down ability, and are believed to kill their target insects by causing changes in the insect cuticle that interfere with the cuticle's desiccation protection properties and/or by suffocation, i.e., blocking the spiracles (Puterka et al. 2003). Inhibition of feeding and oviposition in mites and whiteflies has also been documented (Neal et al. 1994; Liu and Stansly 1995; Slocombe et al. 2008).

Since arabinose, mannose, ribose, and xylose were found to be highly insecticidal to *B. tabaci*, but did not appear to be contact insecticides, experiments were undertaken to determine the mode of action by which these sugars kill *B. tabaci.* Adults were selected as the experimental insects because it is much easier to rear adults than nymphs. The results showed that at the concentrations tested, arabinose, mannose, and xylose did not affect α?-glucosidase and trehalulose synthase activity or the survival of endosymbiont populations in adults (ribose was not tested because its toxicity was not determined until a year after the toxicity of the other test sugars was evaluated). However, arabinose, mannose, and xylose significantly reduced the uptake of radio-labeled sucrose, methionine, and inulin as well as the production of CO_2_ by adults. Ribose also effectively reduced the uptake of radio-labeled sucrose. Therefore, it appears that the insecticidal activity of the sugars is likely due to an antifeedant effect.

The highly toxic sugars acted as strong antifeedants, while of the nontoxic and slightly-moderately toxic sugars that were tested, the relationship in regard to antifeedant activity was not predictable. Thus, in the presence of radio-labeled sucrose, non radio-labeled arabinose, mannose, ribose, and xylose reduced the DPMs in bodies by ≥?77%, while fructose, glucose, galactose, and trehalose significantly reduced the DPMs in bodies by 27–43% (melibiose reduced the DPMs in bodies by 26%, but this value was not statistically significant as compared to the control value). Similarly, the four highly toxic sugars reduced the amounts of radioactivity detected in honeydew by 81–92%, while the moderately toxic sugars melibiose and trehalose and the nontoxic sugar glucose reduced honeydew radioactivity significantly, but to a lesser extent, and nontoxic fructose and mildly toxic galactose did not cause a significant reduction in radioactivity. It is reasonable to suggest that although fructose and glucose reduce feeding, that these sugars at the concentration provided, would not be toxic because the major metabolic pathway for sucrose in *B. tabaci* is its conversion to glucose and fructose (sugars that support growth and development), and thus, *B. tabaci* can tolerate higher concentrations of these two sugars.

To verify the strong antifeedant activity of arabinose, mannose, and xylose additional experiments were conducted in which *B. tabaci* adults were fed radio-labeled methionine or inulin in addition to one of the toxic sugars. Results were similar to those in which *B. tabaci* diets contained radio-labeled sucrose. Because inulin, due to its large molecular size, is not absorbed from the gut and therefore is not metabolized, the most accurate measurement of ingestion was probably provided by the experiments in which radioactivity was measured in the honeydew of *B. tabaci* fed on diets containing radio-labeled inulin and a test sugar. Significantly reduced uptake of both radio-labeled inulin and methionine in *B. tabaci* fed on diets containing arabinose, mannose or xylose further supported the conclusion that antifeedant activity was responsible for the toxicity of the test sugars.

It is not surprising that reduced diet uptake was accompanied by reduced rates of respiration as measured by the production of carbon dioxide. While fructose significantly increased the release of carbon dioxide; arabinose, mannose, and xylose caused large and significant drops in carbon dioxide production as compared to controls. It was unexpected that ribose, one of the most toxic sugars, like melibiose, trehalose, glucose, and galactose, did not cause drops in carbon dioxide production as compared to controls. The stimulatory effect of fructose may be linked to the findings of Salvucci et al. (1999) who reported that at elevated temperatures, *B. tabaci* accumulate sorbitol. There is increased hydrolysis of sucrose to glucose and fructose. Fructose, in turn, can readily be converted to sorbitol, but it is also phosphorylated to produce fructose phosphate and fructose bisphosphate; the latter is a respiratory intermediate.

By definition, an insect antifeedant is a behavior-modifying substance that deters feeding via action on its peripheral sensilla (Kohl 2005). A more liberal definition of antifeedant is: ““any substance that reduces consumption by an insect,”” and would include substances that are ingested or absorbed and would reduce feeding by acting on the central nervous system or exhibit sublethal toxicity to an insect (Isman 2002). It is well-documented that plants produce defensive chemicals, e.g. allomones, that discourage insect feeding (Kohl 2005). Nordlund (1981) defines allomones as substances produced or acquired by an organism which cause a behavioral or physiological response in another species. The allomone adaptively protects the emitter, but not the receiver, and can be highly toxic to the receiver. In addition to plant-produced antifeedant chemicals, it has been reported that organo-metallic compounds and some insecticides reduce insect feeding (Ascher and Rones 1964; Jermy and Matolcsy 1967). Koul (1993) notes that many synthetic compounds could behave as antifeedants for pest insects. Nearly 100,000 compounds have been reported to act as insect antifeedants; 90,100 of them are allomones (Isman 2002). The mechanism of action in antifeedants is, for the most part, unknown (Koul 2005); however, there have been reports of antifeedants, e.g. azadirachtin, stimulating deterrent receptors in a number of herbivorous insects while suppressing sugar and inositol receptors in others (Schoonhoven 1988). The link between stimulation of these receptors and the antifeedant response has not been elucidated, although it has been reported that biogenic amines may be involved (Ikemoto et al. 1995; Omar et al. 1982).

The antifeedant activity of arabinose, mannose, ribose, and xylose to *B. tabaci*, reported here, is insecticidal and the mode of action is not known. It could involve action on the peripheral sensilla of *B. tabaci.* Kennedy and Halpern (1980) reported that feeding deterrents may suppress sugar receptors by changing the activity of these receptors and making them unable to detect sugar, a feeding stimulant. Activity may also be due to interference with the insect's appetite-regulating processes or some other physiological processes. Thus, snowdrop lectin (GNA) which is able to transport neuropeptides from the insect's gut into the hemolymph, when chemically linked to an alatostatin [Manse-AS which inhibits the production of juvenile hormone (JH)] and artificially fed to *Manduca sexta* larvae, caused a severe reduction in feeding and growth (Edwards et al. 2002). Previously, De Wilde (1981) reported that treatment with JH caused changes in insect appetite, and Schmidt et al. (1998) found that an extract of *Melia azedarach* reduced the volume of the JH-producing glands, the corpora allata, of larval *Spodoptera littoralis* and this reduction in size was accompanied by a reduction in hemolymph protein content. It is also possible that the insecticidal substance(s), i.e. the toxic sugars, may compromise an organ or system or interfere with physiological/biochemical process(s) unrelated to the normal regulation of feeding, thus sickening the insect and secondarily causing the insect to stop feeding. Additional studies are necessary to elucidate the mechanism(s) by which arabinose, mannose, ribose, and xylose reduce feeding and kill *B. tabaci.*

Back in 1988, Gatehouse and Hilder noted that economically important crops could be rendered resistant to insect damage by engineering genes encoding elements of pathways for the synthesis of feeding inhibitory compounds. Slocombe et al. (2008) explained that because of the antifeedant, antioviposition, and sometimes toxic properties of the acyl sugars to certain pest species of insects, increasing acyl sugar production has been, and is presently, an important goal of tomato and potato breeding programs. Therefore, these authors undertook and published the results of studies to identify key genes involved in acyl sugar synthesis that were subject to transcriptional control in trichomes of the Solanaceae (Slocombe et al. 2008). It certainly would be worthwhile to capitalize on the antifeedant activity of arabinose, mannose, xylose, and ribose, reported here, to develop genetically engineered host plants (especially ornamental and fiber plants) that would produce sufficient quantities of one or more of the toxic sugars to cause high rates of mortality in *B. tabaci.* Arabinose, mannose, and xylose and thus the genes that regulate their synthesis, have been reported to occur naturally in plants (Burget et al. 2003; Kawasaki 1981; Conklin et al. 1999; Hornung-Leoni 2007). Williams et al. (2000) have provided details about the structure, function, and regulation of monosaccharide and disaccharide transporters and the genes that are involved in their synthesis. In celery, a mannitol synthesizing plant, the mannitol transporter has been identified and characterized (Noiraud et al. 2001). Investigations would also need to be undertaken to determine efficacy, phytotoxicity, and toxicity of the toxic sugars to non-target organisms and the build-up of possible resistance in *B. tabaci.*
